# Effect of a Control Project on Clinical Profiles and Outcomes in Buruli Ulcer: A Before/After Study in Bas-Congo, Democratic Republic of Congo

**DOI:** 10.1371/journal.pntd.0001402

**Published:** 2011-12-27

**Authors:** Delphin Mavinga Phanzu, Patrick Suykerbuyk, Désiré Bofunga B. Imposo, Philippe Ngwala Lukanu, Jean-Bedel Masamba Minuku, Linda F. Lehman, Paul Saunderson, Bouke C. de Jong, Pascal Tshindele Lutumba, Françoise Portaels, Marleen Boelaert

**Affiliations:** 1 General Reference Hospital of Kimpese, Institut Médical Evangélique, Kimpese, Bas-Congo, Democratic Republic of Congo; 2 Department of Microbiology, Mycobacteriology Unit, Institute of Tropical Medicine, Antwerp, Belgium; 3 Central Office of the Rural Health Zone of Kimpese, Bas-Congo, Democratic Republic of Congo; 4 Central Office of the Rural Health Zone of Nsona Mpangu, Bas-Congo, Democratic Republic of Congo; 5 American Leprosy Missions, Greenville, South Carolina, United States of America; 6 Institut National de Recherche Biomédicale, Kinshasa, Democratic Republic of Congo; 7 Department of Public Health, Unit of Epidemiology and Disease Control, Institute of Tropical Medicine, Antwerp, Belgium; Kwame Nkrumah University of Science and Technology (KNUST) School of Medical Sciences, Ghana

## Abstract

**Background:**

Buruli ulcer (BU) is a necrotizing bacterial infection of skin, subcutaneous tissue and bone caused by *Mycobacterium ulcerans*. Although the functional impairment caused by BU results in severe suffering and in socio-economic problems, the disease remains largely neglected in Africa. The province of Bas-Congo in Democratic Republic of Congo contains one of the most important BU foci of the country, i.e. the Songololo Territory in the District of Cataractes. This study aims to assess the impact of a BU control project launched in 2004 in the Songololo Territory.

**Methods:**

We used a comparative non-randomized study design, comparing clinical profiles and outcomes of the group of patients admitted at the General Reference Hospital (GRH) of the “Institut Médical Evangélique” (IME) of Kimpese 3 years before the start of the project (2002–2004) with those admitted during the 3 years after the start of the project (2005–2007).

**Results:**

The BU control project was associated with a strong increase in the number of admitted BU cases at the GRH of IME/Kimpese and a fundamental change in the profile of those patients; more female patients presented with BU, the proportion of relapse cases amongst all admissions reduced, the proportion of early lesions and simple ulcerative forms increased, more patients healed without complications and the case fatality rate decreased substantially. The median duration since the onset of first symptoms however remained high, as well as the proportion of patients with osteomyelitis or limitations of joint movement, suggesting that the diagnostic delay remains substantial.

**Conclusion:**

Implementing a specialized program for BU may be effective in improving clinical profiles and outcomes in BU. Despite these encouraging results, our study highlights the need of considering new strategies to better improve BU control in a low resources setting.

## Author Summary

Buruli ulcer (BU), which is caused by *Mycobacterium ulcerans*, is an important disabling skin disease. However, BU has been neglected in many endemic African countries, including in the Democratic Republic of Congo. The province of Bas-Congo contains one of the most important BU foci of the country, i.e. the Songololo Territory in the District of Cataractes. In 2004 a specialized BU control program was launched in that area. The present study aims to evaluate the impact of the above-mentioned program, by comparing clinical profiles and outcomes of the group of patients admitted at the General Reference Hospital (GRH) of the “Institut Médical Evangélique” (IME) of Kimpese 3 years before the start of the project (2002–2004) with those admitted during the 3 years after the start of the project (2005–2007). The project implementation was associated with a strong increase in the number of admitted BU cases at the GRH and a fundamental change in the profile of those BU patients. Despite these encouraging results, our study provides some limitations of such program, and highlights the need of considering new strategies to better improve BU control in a low resources setting.

## Introduction

Buruli ulcer (BU) is a necrotizing bacterial infection of skin, subcutaneous tissue and bone, caused by an environmental pathogen, *Mycobacterium ulcerans* [1]. Although the functional impairment caused by BU results in severe suffering and in socio-economic problems [2], the disease remains largely neglected by health authorities in Africa [3]. BU is considered as one of the Neglected Tropical Diseases with a poorly known global prevalence [4].

The province of Bas-Congo (Lower Congo) in the Democratic Republic of Congo (DRC) contains one of the most important BU foci of the country, i.e. the Songololo Territory in the District of Cataractes [5]–[10]. Meyers et al. reported that BU existed in that region before 1935 on the basis of interviews of former patients [7]. The first BU case reports were published in the sixties [5]–[7] followed by a long period without reported cases. Since 1999, the general reference hospital (GRH) of the Institut Médical Evangélique (IME)/Kimpese, located in the Songololo Territory, 220 km southwest of Kinshasa, regularly admits BU cases. Between 2002 and 2004 this hospital admitted 64 patients, 95% of them in the ulcerative stage.

During this period, 48 patients out of 64 (75%) were referred by government health centers or other health professionals, 9 (14.1%) by family members, and 7 (10.9%) presented spontaneously. Surgery was the main method of treatment applied amongst these patients (93.7%). An abnormally high case fatality rate (18.7%) was observed among these 64 patients, and whereas 36% presented already a functional limitation at the time of diagnosis, 23% were discharged with permanent disability. The median length of hospitalization was 89 days and, -noteworthy- 90% of the patients were not able to pay their hospitalization costs.

To address these poor clinical outcomes, the American Leprosy Mission and the IME hospital launched a BU control project in Songololo Territory in 2004. The principal aims of this project were (i) the improvement of the patient care of BU patients admitted at the GRH IME/Kimpese and (ii) the promotion of early community-based detection of suspected BU cases. The aim of this study is to evaluate the impact of this specialized BU control program on clinical profiles and outcomes.

## Methods

### Ethics Statement

Ethical clearance for this study was obtained from the Institutional Review Board of IME. All patients, or their guardian in the case of minors, provided informed consent for all diagnostic and treatment procedures and publication of any or all images derived from the management of the patient, including clinical photographs that might reveal patient identity.

The BU control project started at the end of 2004 and introduced free patient care for BU patients during their admission at GRH IME/Kimpese, whereas this was hitherto to be paid on a fee-for-service basis. Furthermore, the patients benefited from a free daily nutritional supplement, and specific antibiotherapy was introduced in accordance with WHO recommendations [11], as well as a physiotherapy program for prevention of disabilities. Simultaneously the project organized awareness raising campaigns in the endemic communities, based on a mass-media approach targeting the general public, followed by active case-finding and referral of suspected cases to the specialized BU care centre. The project was based on the following five components: Improving facilities' management and treatment skills; Prevention of disabilities and physical rehabilitation; Feeding patients and psychological and social support for those affected; Stepping up Information, Education and Communication for the general public and community-based surveillance, and Training and research.

To evaluate the effect of this control project, we used a comparative non-randomized study design, comparing patient demographic profiles and clinical outcomes of the group of patients admitted at the GRH IME/Kimpese in the 3 years before the start of the project (2002–2004) with those admitted during the 3 years after the start of the project (2005–2007).

We have included all consecutive patients clinically diagnosed as BU and admitted to the Surgical Department of GRH IME/Kimpese from January 2002 to December 2007. The clinical case definition elaborated by the World Health Organisation (WHO) was used to diagnose BU [12]. Additionally for the second period, as recommended by the WHO [11], we introduced patients' categorization as follows: A single lesion <5 cm (Category I); A single lesion 5–15 cm (Category II); A single lesion >15 cm, multiple lesions, lesions at critical sites (face, breast and genitalia) or osteomyelitis (Category III). For all patients included in this study, the diagnostic confirmation process consisted of swabs from ulcerative lesions and biopsies for the laboratory confirmation (bacteriology and/or histopathology) of suspected cases according to WHO recommendations [12]. The initial direct smear examinations for acid-fast bacilli and histopathologic analyses were made at the IME/Kimpese laboratory. Other specimens from the same patient were sent in a transport medium to the Mycobacteriology Unit of the Institute of Tropical Medicine (ITM) in Antwerp, Belgium [13], where Ziehl-Neelsen (ZN) staining, in vitro culture on Löwenstein-Jensen medium, and PCR for the detection of *M. ulcerans* DNA were performed according to WHO recommendations [12]. Formalin-fixed tissues were sent to the Department of Infectious and Parasitic Diseases Pathology of the Armed Forces Institute of Pathology in Washington DC, for the histopathological confirmation of diagnosis [10].

Throughout the whole study period, clinical data of BU patients were recorded on standardized Case Report Forms elaborated by WHO (known as form BU01) and the data were entered in a standardized case registry form (BU02) [14]. Next, these data were entered into an Excel database (Microsoft Corporation, Redmond, WA) and analyzed with Epi-Info version 3.3.2 (Centers for Diseases Control and Prevention, Atlanta, GA). The Pearson chi-square test was used to compare proportions with a significance level set at 5%, as well the Fisher's exact test when an expected cell value was less than 5.

To evaluate the relevance and the effect of the BU control project, we used the conceptual framework to evaluate public health programs proposed by Habicht et al. [15]. The principal indicators considered for the data analysis are the number of recorded cases for each period, the number of new cases and relapses, the proportion of cases with functional limitation of joints at diagnosis, the proportion of cases confirmed by at least one laboratory test, the proportion of ulcerative forms at diagnosis, the type of treatment applied, the proportion of discharged cases with functional limitation of joints, the median duration of hospitalization, and the case-fatality rate. Relapse was defined in both study periods as a new confirmed diagnosis of BU less than one year after being declared cured from BU after treatment (surgical only in the first period, antibiotic and/or surgical in the second period). Functional limitation was defined as any reduction in the range of motion of one or more joints, and was assessed based on clinical observation.

Lesions were considered as mixed forms when simultaneous presence of different forms of disease including bone and joint involvement in the same patient was noticed. Besides, we defined as simple ulcerative forms (SUF) the ulcerative lesions not associated with other clinical lesions such as papule, nodule, plaque, edema or osteomyelitis at the same site.

## Results

**Figure 1:**
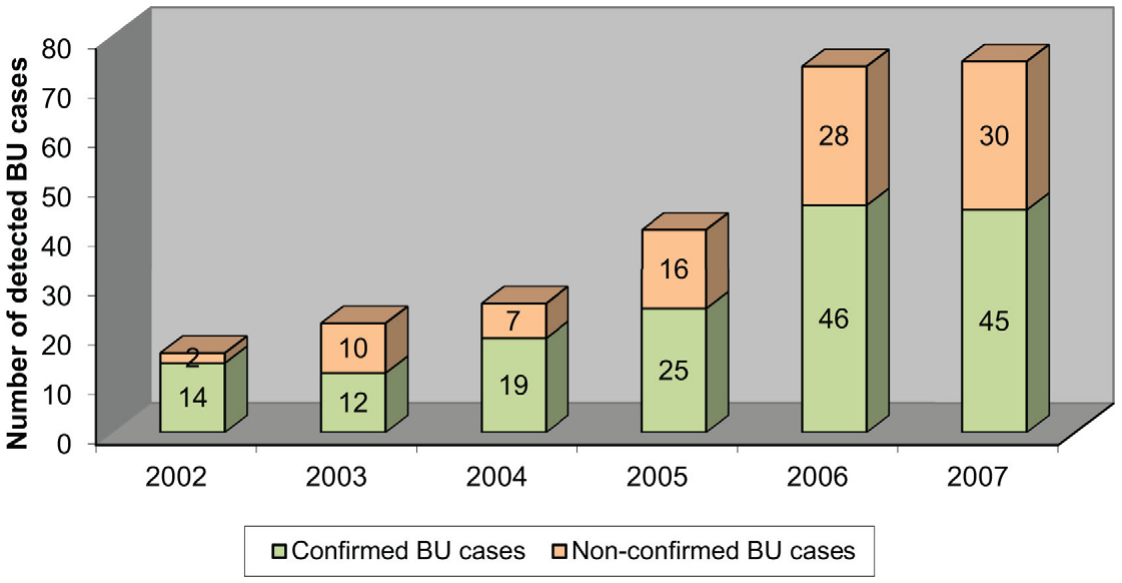
Evolution of number of annual admissions of BU cases to the GRH IME/Kimpese from 2002 to 2007.

**Figure 2:**
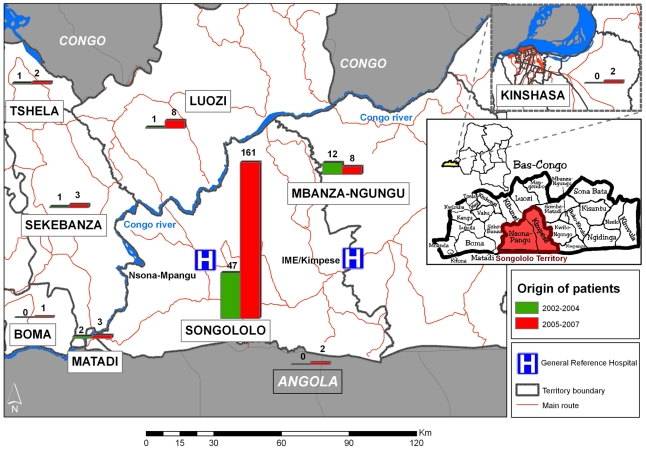
Origin of BU patients admitted in IME/Kimpese Hospital, 2002–2007.

The number of suspected BU cases admitted at GRH IME/Kimpese strongly increased after the start of the BU control project. The average number of annual admissions for BU tripled, from 21 cases per year for the period 2002–2004, to 63 cases per year for 2005–2007 (Figure 1). The clinico-epidemiological features and the results of patient management are shown in Tables 1 and 2. The origin of patients remains mainly the Songololo Territory, Cataractes District, where the GRH IME/Kimpese is located (Figure 2). The median age of patients (20 years) was similar for both periods. The proportion of female patients increased significantly from 30% before to 49% after the project was initiated (p = 0.005).

**Table 1:** The clinico-epidemiological features of BU patients at admission in IME/Kimpese Hospital.

	2002–2004		2005–2007		p
**Number of suspected BU cases**	64		190		
**Average number of annual admissions**	21		63		
**Classification of cases (%)**					
New case	67.2	(43/64)	88.4	(168/190)	<0.001
Relapse	32.8	(21/64)	11.6	(22/190)	<0.001
**Ulcerative forms at detection (%)**	95.3	(61/64)	85.8	(163/190)	0.041
**Clinical Forms (%)**					
Mixed ulcerated forms	64	(41/64)	33.7	(64/190)	<0.001
Simple ulcerated forms	31.3	(20/64)	52.1	(99/190)	0.003
Simple ulcerated forms amongst the ulcers	32.8	(20/61)	60.7	(99/163)	<0.001
Edema	1.5	(1/64)	3.7	(7/190)	0.358*
Nodule	0		2.6	(5/190)	0.231*
Papule	0		0		
Plaque	1.5	(1/64)	2.1	(4/190)	0.628*
Non ulcerative mixed forms	1.5	(1/64)	2.1	(4/190)	0.628*
Suspected osteomyelitis	29.7	(19/64)	14.7	(28/190)	0.007
Confirmed osteomyelitis	14	(9/64)	9.5	(18/190)	0.302
**Sites of lesions (%)**					
Lower limb	65.6	(42/64)	68.4	(130/190)	0.679
Upper limb	35.9	(23/64)	25.8	(49/190)	0.119
Thorax	3.1	(2/64)	2.1	(4/190)	0.471*
Back	4.7	(3/64)	1.6	(3/190)	0.170*
Head and neck	6.3	(4/64)	8.9	(17/190)	0.497
Abdomen	0		1.1	(2/190)	0.558*
Buttock and perineum	0		0.5	(1/190)	0.748*
**Disability at admission (%)**	36	(23/64)	25.8	(49/190)	0.119
**Distribution by age in years (%)**					
≤15	35.9	(23/64)	40	(76/190)	0.564
16–45	37.5	(24/64)	42.1	(80/190)	0.516
>45	26.6	(17/64)	17.9	(34/190)	0.134
**Median age (years)**	19.5		21		
**Sex ratio (M/F)**	2.4/1	(45/19)	1.02/1	(96/94)	
**Proportion of female patients (%)**	30	(19/64)	49	(94/190)	0.005
**Median delay of disease before detection (weeks)**	6		8		

*Fisher exact test (An expected cell value is less than 5).

**Table 2:** Results of the management of BU patients in IME/Kimpese Hospital.

	2002–2004			2005–2007			p
**Healed with disability (%)**							
Amongst all admitted patients	23.4		(15/64)	19.5		(37/190)	0.496
Amongst patients declared cured	31.3		(15/48)	21.0		(37/176)	0.136
**Mode of/State at discharge (%)**							
Death due to BU	18.7		(12/64)	3.2		(6/190)	<0.001*
Healed with complications	23.4		(15/64)	19.5		(37/190)	0.496
Healed without complications	51.6		(33/64)	73.2		(139/190)	0.001
Patients self-discharged	4.7		(3/64)	2.6		(5/190)	0.325*
Transferred	1.6		(1/64)	1.1		(2/190)	0.583*
Patient still under treatment				0.5		(1/190)	
**Laboratory confirmed patients (%)**							
	2002	88	(14/16)	2005	61	(25/41)	0.052
	2003	55	(12/22)	2006	62	(46/74)	0.521
	2004	73	(19/26)	2007	60	(45/75)	0.233
	Total	70	(45/64)	Total	61	(116/190)	0.183
**Treatment applied (%)**							
Rifampin & streptomycin	0		(0/64)	56.3		(107/190)	0
Surgery	93.7		(60/64)	84.2		(160/190)	0.052
Prevention of disability	_			±			
**Median duration of hospitalization (days)**	89			85			
**Case Fatality rate**	18.7		(12/64)	3.2		(6/190)	<0.001*

*: Fisher exact test (An expected cell value is less than 5).

In both periods, the majority of BU patients were new cases, yet the proportion of relapse cases amongst all admissions reduced from 32.8% to 11.6% (p<0.001) after 2004.

**Figure 3:**
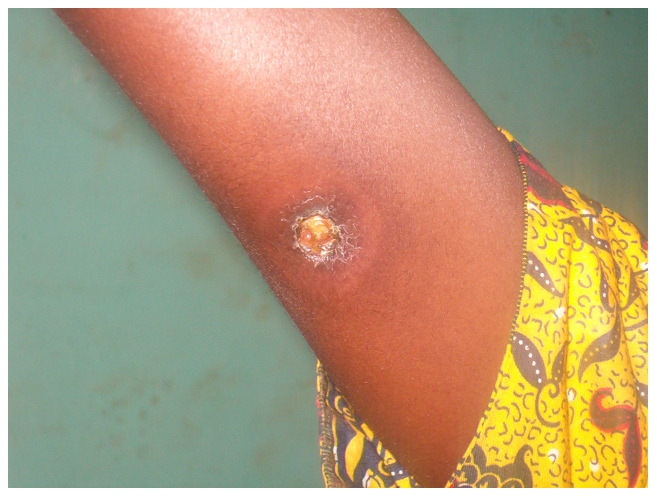
A simple ulcerated form of disease on the right arm.

The proportion of ulcerative forms at admission decreased from 95.3% to 85.8% after 2004 (p = 0.041), and the proportion of SUF increased from 32.8% to 60.7% amongst the ulcers (p<0.001) (Figure 3). There was no change in the proportion of confirmed osteomyelitis nor in the proportion of patients presenting with joint movement limitations. The reported median duration of the disease since the appearance of first symptoms increased from 6 to 8 weeks. Globally, the proportion of patients who healed with complications did not change significantly from 23.4% to 19.5% (p = 0.496), even amongst patients declared cured only, from 31.3% to 21.0% (p = 0.136).

**Figure 4:**
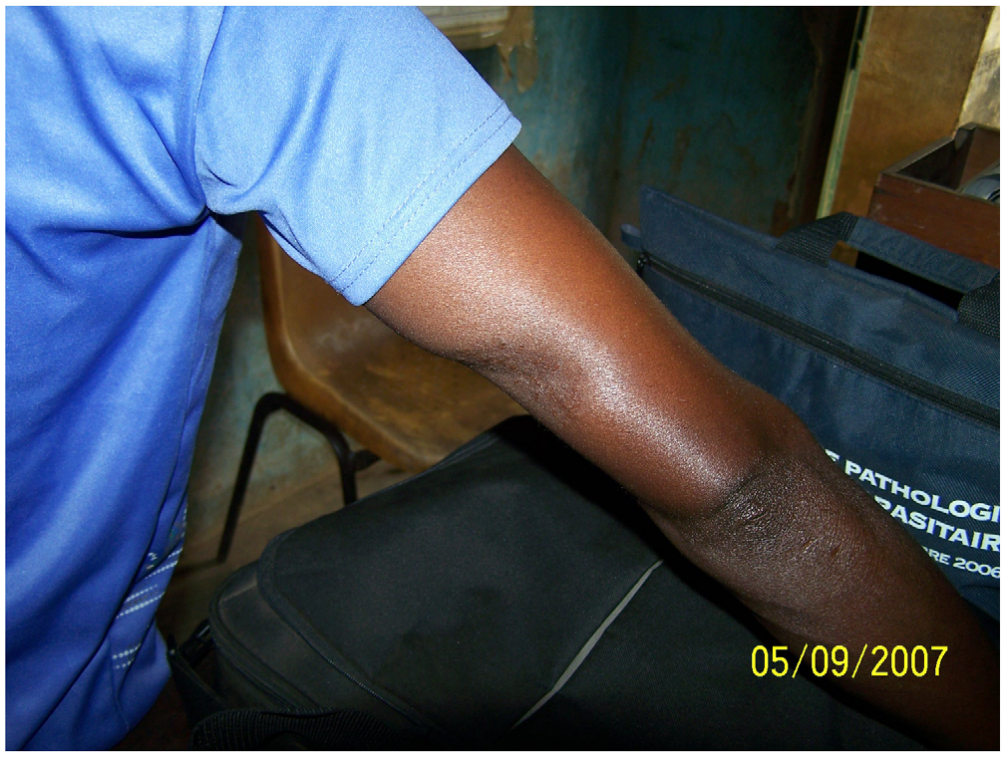
Healing without complications after antibiotherapy combined with surgery.

However, the number of cases that healed without complications increased significantly from 51.6 to 73.2% (Figure 4) (p = 0.001). The proportion of cases confirmed by at least one laboratory test positive for *M.ulcerans* remained the same (70% in 2002–2004 versus 61% in 2005–2007, p = 0.183).

Antibiotic therapy was introduced as part of the control project, and was prescribed to 56.3% of patients, although most patients continued to receive surgery (93.7% previously compared to 84.2% after 2004, p = 0.052). Ninety patients (47.4%) were treated by a combination of antibiotics (rifampicin and streptomycin) and surgery. Seventy patients (36.8%) were treated with surgery alone, seventeen patients (8.9%) only with antibiotics, and thirteen (6.8%) were treated with daily wound dressing.

**Figure 5:**
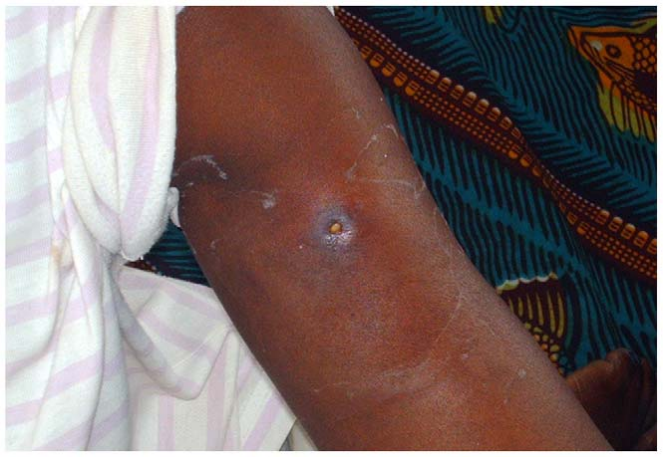
Single ulcerative lesion <5 cm diameter (confirmed by IS*2404*-PCR).

**Figure 6:**
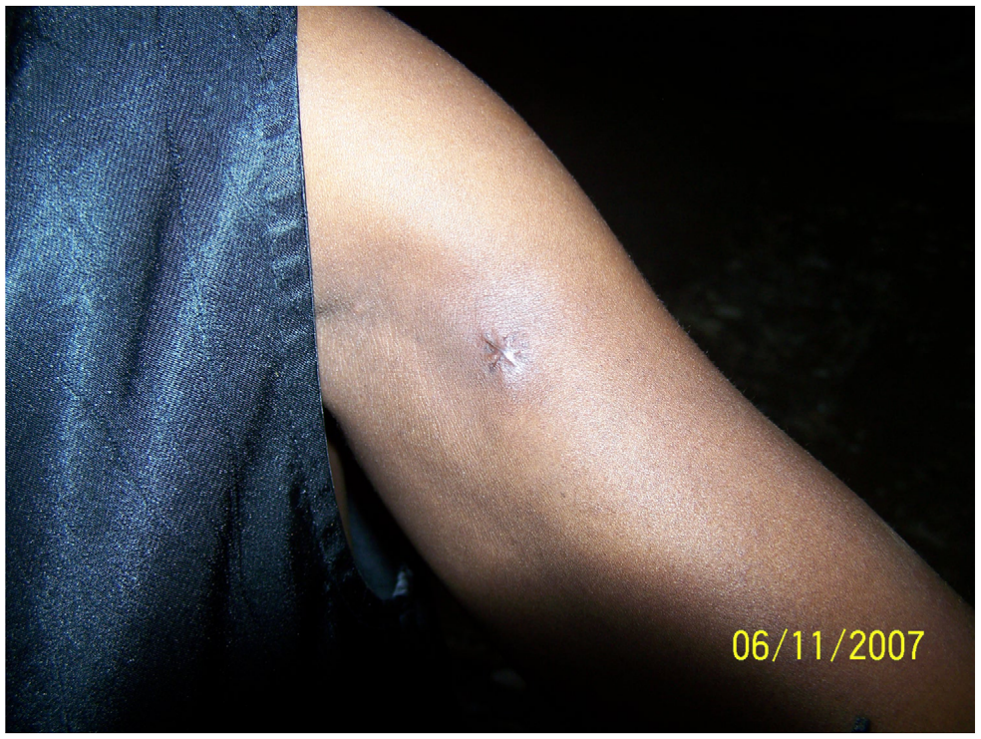
Healed lesion without complication after antibiotherapy alone without surgery.

The median duration of hospitalization, around 90 days, was approximately similar during both periods (Table 2) and varied by disease category during the second period, respectively 60 days for category I (Figure 5 and 6), 81 days for category II, and 118 days for category III.

The case fatality rate was significantly decreased from 18.7% during the previous period (12 out of 64 patients) to 3.2% (6 out of 190 patients) during the second period (p<0.001). Conditions associated with mortality among BU patients in the previous period were as follows: sepsis in four patients out of twelve (33%), malnutrition and anaemia in nine patients (75%), edematous disseminated disease in two patients (16.6%), postsurgical shock in one patient (8%), and cancerization in two patients (16.6%).

## Discussion

The BU control project was associated with a strong increase in the number of admitted BU cases at GRH IME/Kimpese and a fundamental change in the profile of those BU patients. Since the implementation of the control project we observed equal numbers of men and women presenting with BU, significant decrease in the proportion of relapse cases and significant increases in the proportion of early lesions and simple ulcerative forms, and in the proportion of patients healed without complications. Importantly, the case fatality rate decreased significantly from 18.7% to 3.2%.

While those parameters indicate a positive impact of the project, we are aware of the limitations of our study. For our evaluation, we used a historical control group: BU patients admitted at the hospital before the project (2002–2004) were compared to those who benefited from the implementation of the control project (2005–2007). Although such before/after evaluation design does not provide conclusive evidence that the observed changes are attributable to the control project itself, it is usually considered sufficient by policy makers to conclude to a beneficial effect [15]. The threefold increase in the number of BU cases admitted annually can to a large extent be explained by the active case-finding and the reduction of the financial barrier, as patient care was free after 2004, but is probably also due to the improvement of patient management and the quality of clinical results. While both aspects are likely partially involved in the observed results, the observational study design will not allow us to distinguish between the two.

The capacity strengthening of medical staff on the surgical management of BU patients through local and international training, the introduction of specific antibiotherapy (rifampicin and streptomycin), and implementation of a program for the prevention of disabilities have contributed to improvement of clinical outcomes (the increased proportion of patients healed without complications, the reduction of the proportion of relapses, and the reduction of the case fatality rate). Furthermore, we assume that the improved access to adequate and prompt BU treatment in the second period through the free patient care, and the free daily nutritional supplement offered played a major role in the improvement of clinical outcomes during the second period. Indeed, in Africa, the challenge for health care professionals working with BU patients is to break up the cycle of poor clinical outcomes leading to loss of confidence of the affected communities in the hospital [16]. Debacker et al. reported that in the Centre Sanitaire et Nutritionnel Gbemoten (CSNG), Zagnanado, Benin, 68.3% of patients were referred to the hospital by a former BU patient. The improved quality of care at CSNG resulted in a reduction of the median duration of hospitalization from 9 months in 1989 to 1 month in 2001, and the median delay in seeking medical care dropped from 4 months in 1989 to 1 in 2001 [17]. The introduction of a BU program was an important factor in the marked reduction in patient delay. Furthermore, after promotional sessions on BU organized in 2000 by the National BU program in the Zou, Oueme, and Atlantique Departments, patients reported earlier than in 1999 [17]. We are hopeful that similar results will develop at the Territory of Songololo in Bas-Congo.

Awareness raising campaigns followed by active case-finding have contributed to the dissemination of information on BU among the communities in Songololo during the intervention period. We assume that the active case-finding activities have contributed to the change of the Male/Female ratio from 2.4/1 before the project to 1.02/1 during the project period, and thus, the project seems to have contributed to equilibrate the gender balance. During the first period, male BU patients were more frequent probably due to sociocultural barriers for women to seek care, whereas during the second period the active case-finding activities helped the female patients to overcome these barriers.

Progressively, more early lesions and more SUF were diagnosed at the hospital. However, rather surprisingly, the median duration since the onset of first symptoms remained high after the project was launched. Reasons why the median delay in seeking medical attention was higher during the second period compared to the first one remain unclear, and need to be assessed. This may explain the fact that the number of confirmed osteomyelitis cases, limitations of joint movement, both at diagnosis as well as at healing, and patients needing surgery, remained similar. This is problematic, as the huge clinical impact of BU is mainly due to the late detection of cases [18]. Indeed, an extended delay before presentation to the hospital has been identified as one of the most important risk factors for bone involvement. Between 1996 and 2007, out of 930 confirmed and treated BU patients at Zagnanado, Benin, 106 (11.4%) presented an osteomyelitis caused by *M. ulcerans*. The median delay between onset of symptoms and consultation was 167 days for patients with bone involvement and 61 days for those with cutaneous lesions (p<0.001) [19]. In most endemic regions, consulting the hospital seems to be the last resort when other attempts were unsuccessful and when the disease has reached an advanced stage with large cutaneous ulcerations or other complications, such as joint contractures or osteomyelitis [19]. Stienstra et al. reported in their study on the beliefs and attitudes towards BU in Ghana that in 59% of cases, witchcraft was mentioned as cause of the disease. Among the interviewed patients, 52% applied herbs on their lesions and consulted a hospital as last resort. The reasons evoked were (i) financial difficulties [30]% of patients), (ii) the fear of treatment at the hospital and in particular amputation, and (iii) expectations of a spontaneous healing [20]. Recently, a study conducted by Renzaho et al. in Ga West district in Ghana demonstrated that 71.8% of BU patients consulted a traditional practitioner first and that the hospital was consulted as last resort [16]. Meyers and others noted that in the Songololo Territory, DRC, the reasons for which many BU patients delayed seeking medical assistance were obviously complex, but cultural, economic, and transportation factors were especially important [7]. Recently, a study conducted in the same area showed that all interviewed patients first adopted a “wait and see” attitude which lasted on average 2 months [21]. Similar observations were reported in other African countries as Cameroun [22], and Benin [23]–[25]. Those studies were realized when surgery was still the treatment of choice; the recent introduction of specific antibiotherapy as first line treatment may alter this behavior [26]–[28].

These social, economical, geographical and cultural reasons, that limit the access to health care in endemic regions, suggest that the number of admitted patients at GRH IME/Kimpese may represent only the emerged part of the iceberg. The free of charge policy offered to patients does not resolve completely the problem of financial barriers related to the patient management of BU. The study conducted by Grietens et al. in two hospitals with a specialized program for BU in Cameroun, similar to ours, has shown that in spite of the reduction of the treatment costs, the hospitalization for BU remains financially and socially untenable for patients and their households, leading to the abandonment of biomedical treatment or a complete refusal [29]. Therefore, there is a need to consider new control strategies which are both socially and financially acceptable and appropriate for the concerned communities.

### Conclusion

Overall, the results after 3 years of implementation of BU control activities in Songololo Territory are encouraging. However, the morbidity and disabilities due to BU remain high among our patients. The burden of BU in terms of human suffering, long duration of hospitalization, the development of disabling sequelae, and socio-economic repercussions, is mainly attributable to the late detection of cases. For this reason, secondary prevention through earlier case detection and treatment remains one of the key measures in the control of BU [30].

To reduce the burden and to increase the coverage of the population at risk, we consider that a dedicated BU control program at central and provincial level, that operates in close collaboration with the existing polyvalent health services, would be the most efficient way to organize the control of BU in Songololo Territory. The aforesaid program should involve education of the population in the endemic areas, training of healthcare workers, early detection by active case-finding and adequate case management provided free of charge. Further decentralization and integration of BU control activities may improve access to diagnosis and care at the most peripheral level of the health system. A close collaboration between the BU control project and the health zones is essential for the implementation of a simple, functional, and efficient active surveillance system in a resource-limited context.
